# Holocord syringomyelia in 18 dogs

**DOI:** 10.3389/fvets.2024.1514441

**Published:** 2025-01-08

**Authors:** Despoina Douralidou, Lorenzo Mari, Sophie Wyatt, Juan Jose Minguez, Patricia Alvarez Fernandez, Carlo Anselmi, Christoforos Posporis

**Affiliations:** ^1^The Ralph Veterinary Referral Centre, Marlow, United Kingdom; ^2^Queen Mother Hospital for Animals, Royal Veterinary College, Hatfield, United Kingdom; ^3^Pride Veterinary Referrals, IVC Evidensia Group, Derby, United Kingdom; ^4^Blaise Veterinary Referral Hospital, IVC Evidensia Group, Birmingham, United Kingdom

**Keywords:** myelopathy, Chiari-like, malformation, French bulldog, tetraparesis

## Abstract

Holocord syringomyelia (HSM) is characterized by a continuous spinal cord cavitation along its entire length and is currently poorly documented in dogs. This retrospective multicentric case series investigates the clinical and MRI findings in 18 dogs with HSM. The median age at presentation was 82 months (range 9–108 months) and French Bulldogs were overrepresented (50%). Signs of myelopathy attributed to HSM were detected in 12 dogs (67%), spinal pain/paresthesia/allodynia in eight (44%), and four dogs (22%) had no HSM-associated signs. The most common neuroanatomical localization was C1-C5 spinal cord segments. On MRI, the cranial limit of HSM was at C1 vertebra in seven (39%) and at C2 in 11 dogs (61%). The caudal limit extended to L3 in four dogs (22%), L4 or L5 in six dogs (33%) each, and L6 in two dogs (11%). The conus medullaris terminated at L5 in one dog (6%), L6 in 11 (61%), and L7 in six dogs (33%). Seventeen dogs (94%) exhibited MRI features of Chiari-like and/or other intracranial/cranio-cervical junction malformations. One dog (6%) was treated surgically, 11 (61%) medically, and six (33%) received no treatment. Over a median follow-up of 12 months (range 2–65 months) in 16 dogs, one dog (6%) improved, nine (56%) deteriorated, four (25%) were static, and two (13%) remained free of HSM-associated signs. This is the first study to describe canine HSM, highlighting a possible breed predisposition in French Bulldogs. The frequent occurrence of myelopathy and disease progression underscores the need for further research into the underlying etiopathogenesis, natural disease evolution, and response to therapeutic interventions.

## Introduction

Syringomyelia (SM) is a common spinal cord condition predominantly diagnosed in small breed dogs such as the Cavalier King Charles Spaniel (CKCS), Griffon Bruxellois, Pomeranian, Chihuahua, and French Bulldog (FB) (1–8). It is described as a fluid-filled cavitation of one or more segments of the spinal cord parenchyma (9) and has traditionally been associated with Chiari-like malformation (CM) in dogs (9, 10).

More recent insights into the etiopathogenesis of canine SM suggest the involvement of complex cranio-cervical junction and skull malformations associated with brachycephaly and neural parenchymal displacement. This is comparable to complex craniosynostosis syndromes in people (11, 12). While the etiology of SM remains complex and not fully elucidated, it is generally believed that disturbed dynamics of cerebrospinal fluid (CSF) circulation may play a key role (9, 10, 12). Neoplastic, inflammatory, anomalous, and traumatic conditions have also been associated with SM (13–17).

Dogs with SM can develop clinical signs suggestive of neuropathic pain and/or paresthesia as the result of gray matter dorsal horn damage and subsequent abnormal processing of sensory information (9, 18, 19). In CKCS, while spinal pain has been reported with CM even in the absence of SM, signs of myelopathy were only seen in dogs with a transverse syrinx width of 4 mm or more (20).

Holocord SM (HSM) has been described as “joining up” of cervical, thoracic, and lumbar syringes leading to a continuous parenchymal cavitation of affected spinal cord segments, typically extending from C2 to L4 (21). This definition implies that HSM may represent a progression of multifocal SM, a possibility that has indeed been documented in one dog (11). A study investigating the distribution of SM in CKCS reported that 76% of dogs with SM within the C1-C4 region also had SM within the C5-T1 and/or T2-L2 segments, but not in a continuous pattern. In the same study, a lower percentage of dogs had syringomyelia in the L3-L7 region (1). Nevertheless, longitudinal studies with sequential MRI are yet to confirm the progression of multifocal SM into the holocord variant as a morphopathological evolution of the condition (22). It is also our experience that HSM is diagnosed at first presentation in most cases. As a result, the exact cause of HSM and its evolution over time remain unknown.

In people, HSM is rare and has been reported in association with spinal cord neoplasms (23), trauma (24), Chiari and other intracranial malformations (25, 26). There is currently no published data focusing specifically on HSM in dogs. Its presenting neurological and MRI features as well as its clinical progression and response to different therapeutic interventions are yet to be investigated. As a result, prognostication remains challenging which makes decision-making difficult in clinical practice. The aim of our study is to describe the presenting clinical signs, MRI findings and outcome in a series of dogs with HSM.

## Materials and methods

This retrospective descriptive multicentric case-series was conducted using the clinical databases of three United Kingdom referral hospitals to identify dogs diagnosed with HSM. Ethical approval was granted by the Social Science and Ethical Review Board of the Royal Veterinary College (URN SR2022-0089) and all selected patients had informed consent from owners for participation in the study.

Dogs of any age, sex, breed, and neuter status were included if they met the following criteria: (a) full access to complete clinical records; (b) access to MRI study with a minimum of sagittal T2w images covering all spinal cord segments; (c) diagnosis of HSM on MRI, as assessed by a board-certified radiologist or neurologist, and defined as a continuous intramedullary cavitation involving cervical, thoracic and lumbar spinal cord segments in a continuous pattern with isointense signal to normal CSF. Cases with incomplete medical records and/or neurological assessment were excluded.

Detailed information regarding signalment, clinical history and presenting complaint, physical and neurological examination, MRI findings, results of additional diagnostic tests, treatment, and outcome was retrieved from medical records. The age at onset of clinical signs, age at presentation, sex, and neuter status were noted. Clinical history included information about the duration and type of clinical signs prior to presentation, response to previous therapeutic interventions, and any relevant data about concurrent conditions.

Neurological examinations were performed by board-certified or residency-trained board-eligible neurologists. Neurological signs consistent with myelopathy included the presence of general proprioceptive ataxia, motor impairment (paresis or paralysis), with postural reaction deficits involving one or more limbs. Spinal pain was defined as hyperesthesia to palpation of the cervical, thoracic, lumbar and/or lumbosacral region/s. Other signs of possible neuropathic pain/paresthesia/allodynia were assessed individually by the responsible clinician and included but were not limited to spontaneous/postural/action-related vocalization (e.g., when scratching the cervical region, when moving head), an abnormal head or neck restrictive posture that could not be explained by another condition, licking a distal limb in the absence of dermatological or orthopedic disease, aversion to touch, scratching/rubbing the head, ears and/or neck in the absence of dermatological disease, and phantom scratching (not making contact with skin while scratching) (20).

A neurological grading system, adapted from the modified Frankel scale for osseous-associated cervical spondylomyelopathy (27) and from a neurological scale grading thoracolumbar spinal cord injury (28), was used to categorize the severity of neurological impairment (Table 1). Neuroanatomical localization was recorded, with spinal cord segments divided into C1-C5, C6-T2, T3-L3, and L4-S3 regions. Intra-cranial, cauda equina, vestibular apparatus, and/or peripheral nervous system neuro-anatomical localizations were also noted when present. Additional data focused specifically on the presence of lateralization of neurological signs, and central cord syndrome. Central cord syndrome was defined as significantly more severe paresis affecting the thoracic limbs compared to pelvic limbs secondary to a C1-T2 myelopathy (29).

**Table 1:** Modified neurological grading scale (27, 28).

Grade	Neurological signs
0	No neurological deficits or spinal pain/paresthesia/allodynia
1	Spinal pain/paresthesia/allodynia without neurological deficits
2	Ambulatory tetraparesis/hemiparesis or paraparesis/monoparesis with or without spinal pain/paresthesia/allodynia
3	Non-ambulatory tetraparesis/hemiparesis/paraparesis with or without spinal pain/paresthesia/allodynia
4	Tetraplegia or paraplegia with or without spinal pain/paresthesia/allodynia
5	Tetraplegia with ventilatory compromise or paraplegia with loss of deep pain perception with or without spinal pain/paresthesia/allodynia

Magnetic resonance imaging (MRI) was performed using 1.5 Tesla Magnets (Intera and Ingenia CX, Philips Medical Systems, Eindhoven, Netherlands; Siemens Magnetom Essenza, Frimley, United Kingdom; HDe, GE Healthcare, United Kingdom; Signa Explorer GE Healthcare, United Kingdom). MRI sequence and plane selection varied depending on different protocols used at each referral hospital and imaging requirements of each case. These included a variable combination of T2-weighted, short tau inversion recovery (STIR), fluid attenuated inversion recovery (FLAIR), gradient echo, pre- and post-contrast T1-weighted sequences in sagittal, transverse, and/or dorsal planes. Post-contrast images were acquired when deemed necessary on an individual basis using gadoterate meglumine (Dotarem R, Guerbet, United Kingdom; Clariscan, GE Healthcare, Australia).

Syringomyelia was defined as a fluid-filled intramedullary cavitation with MRI signal isointense to normal CSF (T2-weighted/STIR strongly hyperintense, T1-weighted strongly hypointense, suppressed on FLAIR images, without contrast enhancement) (30). The holocord variant of SM was defined as an extensive, continuous syrinx spanning the cervical, thoracic, and lumbar spinal cord segments, appearing uninterrupted on sagittal T2-weighted images (21). The cranial and caudal limits (vertebral segment) of HSM were recorded, as well as the level of conus medullaris termination. HSM was characterized as uniform when the syrinx height on mid-sagittal T2-weighted images appeared unchanged along the spinal cord. Non-uniform HSM indicated an evident change of the syrinx height in at least one spinal cord region as seen on mid-sagittal T2-weighted images. The presence of skull, cranio-cervical junction, Chiari-like, intra-cranial neural parenchyma malformations, or any additional potentially significant structural changes were noted based on definitions from previous descriptions (12). The severity of CM was rated based on a modified grading scale adapted from Wijnrocx and colleagues (31) (Table 2). Lastly, the presence of CSF flow artifact observed on MRI as T2-weighted hypointense and/or FLAIR-hyperintense signal (32) within any segment of HSM, subarachnoid space, and/or in the ventricular system was reported.

**Table 2:** Modified CM severity scale (31).

Grade	MRI findings
0	No supraoccipital cerebellar indentation or cerebellar vermis foramen magnum impaction/herniation
1	Supraoccipital cerebellar indentation with visible CSF signal between cerebellum and brainstem (no cerebellar vermis foramen magnum impaction/herniation)
2	Cerebellar vermis impaction into the foramen magnum
3	Cerebellar vermis herniation through the foramen magnum
4	Severe cerebellar vermis herniation through the foramen magnum with tongue-shaped appearance

Additional diagnostic tests were performed when deemed necessary and included hematology, serum biochemistry, urinalysis, CSF analysis, gluten-sensitivity serological testing, total T4 and TSH, screening tests for infectious and neurodegenerative diseases, radiography, and CT scan of other body regions. CSF protein concentration > 30 mg/dL for cerebellomedullary and > 45 mg/dL for lumbar sampling and total nucleated cell count (TNCC) > 5 cells/μl were classified as increased (33).

The presence of concurrent diagnoses was documented for each case and HSM was classified as incidental or clinically significant as per the data from clinical history, neurological examination, and investigations. Clinical signs believed to be associated with concurrent diagnoses were separately documented. The potential association between HSM and any co-existing non-developmental intra-cranial or spinal pathology identified on MRI was investigated. The prevalence of myelopathic or pain-related signs attributed to HSM was calculated after exclusion of other possible causes with strict consideration of concurrent diagnoses.

Treatment for HSM was divided into medical, surgical, or no treatment. Medical therapy was further characterized as monotherapy when a single medication was used or polytherapy when a combination of two or more medications was given. The number of dogs receiving anti-inflammatory medications, non-steroidal or corticosteroids, was also reported together with the number of dogs receiving gabapentinoids.

Follow-up information was obtained by accessing clinical records of re-examinations and telephone updates, and by completion of a non-validated but standardized questionnaire by owners (Supplementary materials) adapted from previous descriptions in veterinary literature (34). Video recordings were also requested and assessed if available. Follow-up period was defined as the number of months from MRI diagnosis until the last communication with the owner as assessed from clinical notes and from follow-up questionnaires. The overall clinical course of HSM and the evolution of specific HSM-associated clinical signs were investigated at last follow-up based on clinical notes from repeated neurological examinations and additional information obtained from telephone updates, video footage, and completed questionnaires. The overall quality of life (QoL) was subjectively assessed by owners as part of the standardized questionnaire. Owner-reported QoL was rated on a scale from one to five, one being very poor and five excellent.

## Results

### Case recruitment

Clinical records from all participating hospitals were reviewed to identify dogs with HSM by assessing cases with reported extensive SM. Of 51 identified cases, eight were excluded due to incomplete MRI studies that did not cover the entire spinal cord, and 25 because the condition lacked a continuous holocord pattern across all spinal cord regions. No incomplete medical records were identified. Among the excluded cases, there were 12 CKCS, 11 FB, three Pomeranians, two Yorkshire Terriers, two crossbreeds, one Miniature Pinscher, one Miniature Poodle, and one Boston Terrier. Eighteen dogs met all study criteria and were included in the cohort for descriptive analysis.

### Signalment

The median age at presentation was 82 months (range 9–108 months), and the median body weight was 10kg (range 2–26kg). Eleven (61%) dogs were male (seven intact, four neutered) and seven (39%) were female (three intact, four neutered). French Bulldogs were overrepresented with nine cases (50%), followed by Chihuahuas with three (17%), Boston Terriers with two (11%), CKCS with two (11%), Staffordshire Bull Terriers with one (6%) and crossbreed with one (6%). The average prevalence of FB in the general canine hospital population across the three participating referral hospitals was 4.8%.

### Clinical history and examination

Nine dogs (50%) were presented for unrelated conditions, including episodes of severe lumbosacral pain (one dog), gastrointestinal signs (one dog), generalized tonic–clonic epileptic seizures (three dogs), myoclonic episodes (one dog), paroxysmal dyskinetic events (one dog), acute onset thoracic limb lameness after a fall (one dog), and acute painful pelvic limb lameness/monoparesis (one dog). Neurological assessment and clinical history suggested that five of these dogs had concurrent signs relating to HSM. The other four reached a diagnosis of HSM incidentally during diagnostic imaging investigations for their concurrent conditions and were not deemed to have HSM-related clinical signs. The remaining nine cases in our cohort were referred with history of chronic tetraparesis in six, with (3 dogs) or without (3 dogs) pain-related behaviors, thoracic limb weakness in one, and mild ambulatory paraparesis in two.

The median duration of clinical signs prior to presentation was 7 months (range 2–102 months), derived from 11 cases. Based on clinical history and examination findings, the presence of HSM-associated spinal pain/paresthesia/allodynia and signs of myelopathy, the neurological grades, and neuroanatomical localizations are demonstrated in Table 3. The following combinations of HSM-related signs were observed: six dogs (33%) exhibited myelopathy alone, two (11%) had spinal pain/paresthesia/allodynia alone, and six (33%) displayed both myelopathy and spinal pain/paresthesia/allodynia. Four dogs showed no clinical signs attributable to HSM and were classified as clinically normal for HSM, with the remaining 14 (78%) having clinically significant HSM. A focal neuroanatomical localization was determined in nine dogs and multifocal in seven. The most common neuroanatomical localization was C1-C5 in 11 dogs (Table 3). Mild lateralization of neurological deficits was reported in six dogs, while none had central cord syndrome.

**Table 3:** Neurological signs attributed to HSM and concurrent diagnoses at presentation in 18 dogs.

Case no.	HSM-associated myelopathy (Yes/No)	HSM-associated neuropathic pain/paresthesia/allodynia (Yes/No)	HSM-associated neurological grade (0–5)	Neuroanatomical localization	Concurrent diagnoses^§^	Clinical signs of concurrent diagnoses*
1	Yes	Yes	2	C1-C5	–	–
2	Yes	Yes	2	C1-C5	–	–
3	Yes	No	2	C1-C5 Cauda equina Right vestibular apparatus	L7-S1 IVDE Previous otitis	Episodic lumbo- sacral pain^**^ Right head tilt
4	Yes	No	2	C1-C5 T3-L3	Gastric FB	Vomiting and diarrhea
5	Yes	No	2	C1-C5 T3-L3	–	–
6	No	No	0	Forebrain	IE	GTCS
7	Yes	Yes	2	C1-C5	–	–
8	Yes	Yes	2	C1-C5	–	–
9	Yes	Yes	2	C1-C5	–	–
10	Yes	No	2	T3-L3	–	–
11	No	Yes	1	C6-T2 T3-L3 Forebrain	Idiopathic epileptic myoclonus	Episodic myoclonus of the head, neck, and thoracic limbs
12	Yes	No	2	Forebrain C1-C5	PD	Self-limiting episodes of dystonia
13	No	No	0	-	OA and left bicipital tendinopathy	Left thoracic limb lameness
14	Yes	No	2	Forebrain C1-C5	IE	GTCS
15	No	No	0	Forebrain	IE	GTCS
16	No	No	0	L4-S3	Lateralized L5-L6 IVDE	Left pelvic limb lameness-monoparesis^⊥^
17	No	Yes	1	Undetermined^^^	Mild hypertrophic C2 ganglioneuritis	Episodic non-specific spinal pain^^^
18	Yes	Yes	2	C1-C5 Right vestibular apparatus	Suspected fourth ventricle diverticulum	Right head tilt
**Prevalence**	67%	44%	Grade 0: 22% Grade 1: 11% Grade 2: 67%	–	61%	–

IVDE: intervertebral disk extrusion; FB: foreign body; IE: idiopathic epilepsy; GTCS: generalized tonic clonic seizures; PD: paroxysmal dyskinesia; OA: osteoarthritis. ^§^Incidental imaging abnormalities with no associated clinical signs are not listed. *Clinical signs of concurrent diagnosis not considered for neurological grading of HSM. **Lumbosacral pain unrelated to HSM. ⊥Pelvic limb monoparesis unrelated to HSM. ^ Abnormal behavior characterized by spontaneous vocalization that could not be localized on examination (forebrain vs. spinal pain-related) and was attributed to either or both HSM and concurrent diagnosis.

Eleven (61%) dogs were receiving treatment at the time of presentation, which included an NSAID alone (three dogs), an NSAID with paracetamol and gabapentin (one dog), gabapentin alone (two dogs), NSAID with gabapentin (one dog), gabapentin with paracetamol (one dog), oclacitinib with omeprazole, sucralfate and vitamin B12 (one dog), phenobarbital with levetiracetam (one dog), and diazepam (one dog). Eight of these dogs were on treatment for signs of suspected neuropathic pain/allodynia/paresthesia out of which three were reported to have shown a partial improvement, one had deteriorated, and four had remained static at presentation.

### Investigations

**Figure 1:**
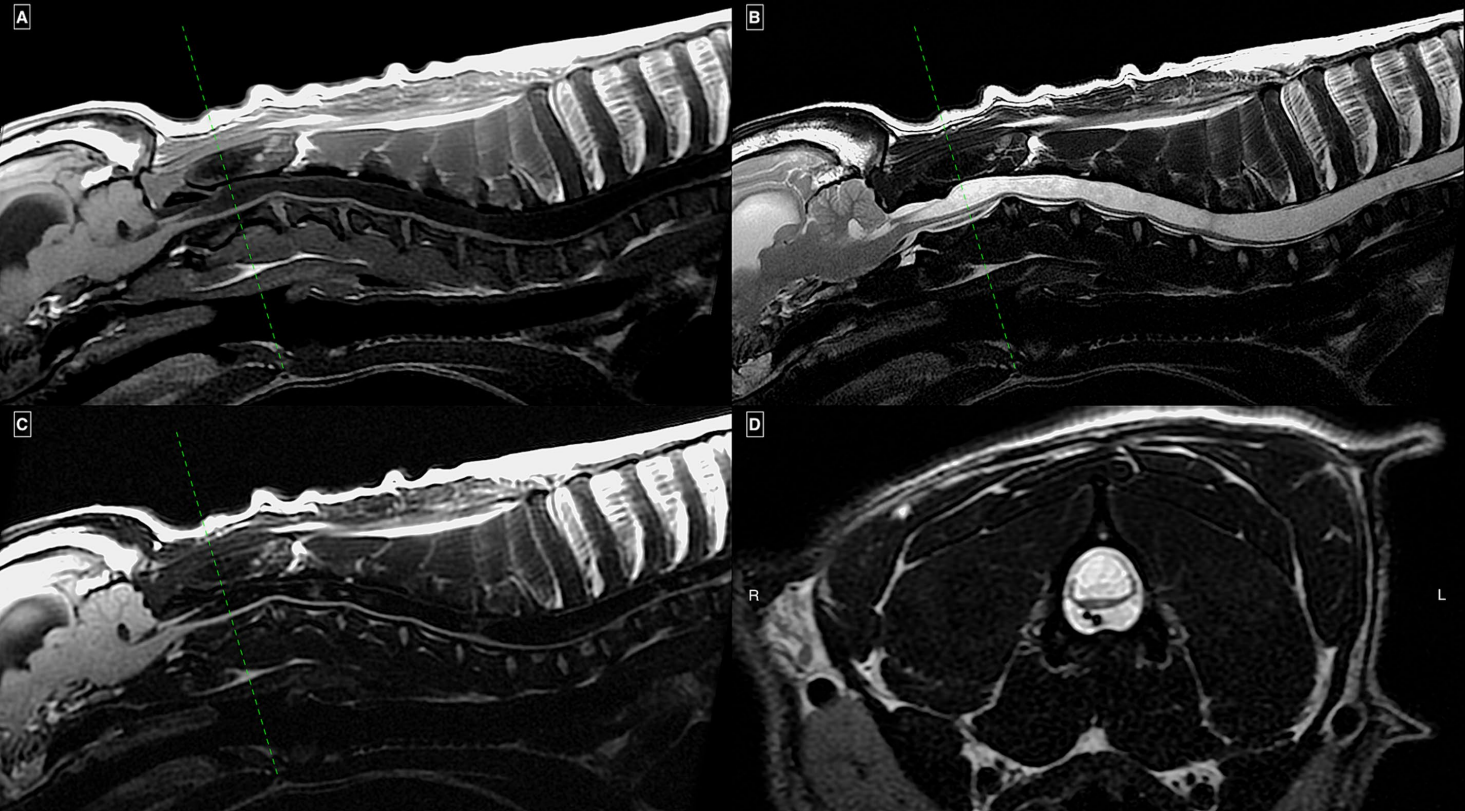
MRI study of a French Bulldog with HSM on mid-sagittal T1w **(A)**, T2w **(B)**, and FLAIR **(C)** images of the cervical and cranial thoracic spinal regions together with a transverse T2w image **(D)** at the level of mid-C2 vertebral body. There is an extensive continuous homogeneous T2w hyperintense and T1w/FLAIR hypointense intramedullary well-demarcated cavitation of the included spinal cord segments occupying more than 75% of its transverse diameter. Enlargement of the visible lateral ventricle and mild impaction of the cerebellum into the foramen magnum (“coning”) can also be seen.

Magnetic resonance imaging of all spinal cord segments was performed on various sequences and planes (Figures 1^2^). MRI protocols included sagittal T2W images of all segments in all cases. Twelve dogs had T1W images acquired in at least one region, most commonly the cervical. Seven dogs had STIR images performed in one region, and three had FLAIR. Other less frequently acquired sequences included T1W post contrast, dorsal T2W, Volume Isotropic Turbo Spin Echo Acquisition (VISTA) and Balanced Turbo Gradient Echo (BALTI). An MRI study of the head was available in 16 dogs on a minimum of sagittal T2W sequence. Eight of these cases had additional T2W images on different planes, Gradient echo, FLAIR, and pre- and post-contrast transverse T1W images.

**Figure 2:**
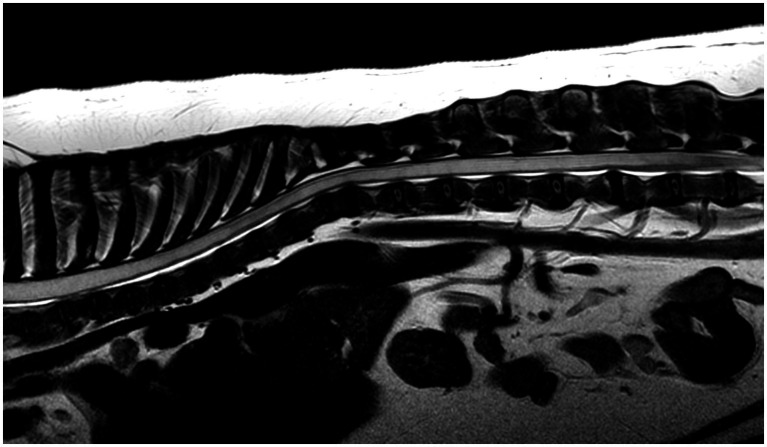
MRI study of a French Bulldog with HSM on a mid-sagittal T2w image of the thoracic and lumbar spinal regions showing an extensive severe homogeneous T2w hyperintense intra-medullary well-demarcated cavitation involving most visible spinal cord segments in a continuous pattern, terminating at L5 vertebral body. There is also multifocal intervertebral disk degeneration and non-compressive thoracic vertebral malformations.

The cranial limit of HSM was at C1 vertebra in seven dogs (39%) and at C2 in 11 (61%). The caudal limit extended to L3 in four dogs (22%), L4 or L5 in six dogs (33%) each, and L6 in two dogs (11%). The conus medullaris terminated at L5 in one dog (6%), L6 in 11 (61%) and L7 in six dogs (33%). HSM was characterized as uniform in five (28%) and non-uniform in 13 cases (72%). CSF flow artifact was suspected in 14 dogs (78%).

Seventeen (94%) dogs exhibited MRI features of CM and/or other intracranial/craniocervical junction malformations. Cerebellar morphology and vermis position in relation to skull structures and craniocervical junction were possible to assess in 17 dogs. Overcrowding of the caudal cranial fossa was detected in 15 dogs (88%). Using our modified CM grading system, seven dogs (38%) were scored as grade 1, four (22%) as grade 2, three (17%) as grade 3, and two (11%) as grade 4, whereas two dogs (11%) had no CM (grade 0). The presence of ventriculomegaly was possible to assess in 16 cases and was detected in 15 (94%). Of 15 dogs assessable for other brain malformations, six had supracollicular fluid accumulation, and one a suspected fourth ventricle arachnoid diverticulum. One dog with supracollicular fluid accumulation also had absent septum pellucidum. Five of the six dogs with supracollicular fluid accumulation and the dog with fourth ventricle arachnoid diverticulum had concurrent ventriculomegaly.

Only one dog (6%) had atlanto-occipital overlap and three (17%) displayed increased odontoid angulation with elevation of the craniocervical junction (medullary kinking). Eight dogs (44%) had dorsal atlanto-axial bands, three of which were compressive. Concurrent spinal pathology was observed in 14 dogs (78%) and included: mildly compressive to non-compressive intervertebral disk protrusion (10 dogs), thoracic hemivertebrae with or without mild non-compressive kyphosis (seven dogs), mildly compressive intervertebral disk extrusion (one dog), transitional vertebra (one dog), chronic focal intramedullary changes at C6 from a previous spinal cord injury (one dog), spondylosis deformans (one dog), mildly compressive hypertrophic C2 ganglioneuritis (one dog), and mild left sided non-compressive articular facet hypertrophy at T3-T4 (one dog) causing epidural fat effacement.

One dog showed mild abnormalities on hematology and serum biochemistry, linked to gastro-intestinal disease (low albumin, elevated ALP, ALT, and GLDH). Gluten sensitivity testing in a dog with paroxysmal dyskinesia revealed elevated anti-transglutaminase IgA. Thyroid function, genetic testing for neurodegenerative conditions, and infectious disease screening were unremarkable. CSF analysis in four dogs revealed mild mononuclear pleocytosis in two with normal protein levels (TNCC = 7 cells/μl; TNCC = 6 cells/μl). Additional imaging findings included a heart-base mass, segmental megaesophagus with aspiration pneumonia, gastroenteritis confirmed as lymphoplasmacytic gastritis and duodenitis on biopsies, degenerative joint disease with bicipital tendinopathy, and normal hip radiographs in one dog each.

### Diagnosis

Based on data from clinical history, examination, and diagnostic investigations, HSM was considered responsible for some or all clinical signs in 14 dogs (78%). Other clinically relevant concurrent diagnoses and associated clinical signs are listed in Table 3.

### Treatment

Twelve dogs (67%) received treatment for HSM following diagnosis, four (25%) were managed solely for other conditions, and two (11%) with HSM-related signs but no pain/paresthesia/allodynia received no treatment. One dog underwent a surgical intervention for CM-HSM combined with medical therapy, while 11 were managed conservatively. Medical treatments for HSM included gabapentin (11 dogs), prednisolone (7 dogs), NSAIDs (1 dog), paracetamol (2 dogs), and amitriptyline (1 dog), either as polytherapy in seven dogs or monotherapy in four. The dog treated surgically underwent caudal cranial fossa decompression after a failed six-month trial of prednisolone and gabapentin associated with mild neurological deterioration. The procedure involved a suboccipital craniectomy, excision of a hypertrophic atlanto-occipital membrane, and dural marsupialization. A C2-C3 ventral slot was planned but mislocalization led to an intervention at C3-C4 intervertebral disk space, confirmed by post-operative MRI. The initially intended C2-C3 ventral slot was performed 6 days later.

### Follow-up and outcome

Follow-up information was available in 16 dogs (median = 12 months, range 2–65 months). Two dogs were reported deceased with no cause of death specified. All 16 remaining dogs were alive at the time of writing. Follow-up information was collected through completed questionnaires and telephone updates for all 16 dogs, supplemented by video recordings for 10 dogs and full re-examinations by a neurologist for the remaining six.

Owner-rated QoL was excellent (grade 5) in six dogs, grade 4 in four dogs, grade 3 in three dogs, and grade 2 in three dogs. The overall clinical course, neurological grade, evolution of neuropathic pain/paresthesia/allodynia and signs of myelopathy are documented in Table 4.

**Table 4:** Clinical course of neurological signs attributed to HSM at follow-up.

Case No.	Time of follow-up (months)	Signs of myelopathy*	Signs of spinal pain/paresthesia/allodynia*	Neurological grade	Overall clinical course of HSM-associated clinical signs*	Medical treatment for HSM (Yes/No)
1	17	Static	Static	2	Static	No
2	17	Deteriorated	Deteriorated	2	Deteriorated	Yes
3	11	Deteriorated	Deteriorated^	2	Deteriorated	Yes
4	13	Deteriorated	Deteriorated^	2	Deteriorated	Yes
5	6	Static	No signs	2	Static	No
6	5	No signs	Deteriorated^	**1**	Deteriorated	Yes
7	11	Deteriorated	Static	2	Deteriorated	Yes
8	20	Improved**	Improved**	2	Improved**	Yes
9	14	Static	Deteriorated	2	Deteriorated	Yes
10	18	Deteriorated	Deteriorated^	2	Deteriorated	Yes
11	2	No signs	Static	1	Static	Yes
12	2	Deteriorated	No signs	2	Deteriorated	Yes
13	8	No signs	No signs	0	No signs	No
14	10	Static	No signs	2	Static	Yes
15^⊥^	–	–	–	–	–	–
16	65	No signs	No signs	0	No signs	Yes
17	40	Deteriorated^	Deteriorated	**2**	Deteriorated	Yes
18^⊥^	–	–	–	–	–	–

*Clinical course characterized as “improved/deteriorated/static” or as “no signs” at the time of diagnosis or follow-up. **Treated surgically. ^No signs at presentation but present at follow-up. Bold font indicates change (deterioration) in neurological grade at follow-up. ⊥Deceased at the time of follow-up.

Thirteen dogs (81%) were on medical treatment, including the one who had previously undergone surgery for CM-HSM. Prednisolone was used in six cases, gabapentin in 11, paracetamol in two, amitriptyline in one, and NSAID in four, either as polytherapy in nine dogs or monotherapy in four. Of the 12 dogs treated at diagnosis, 11 were still on treatment for HSM, and one was off medication at follow-up. Additionally, two dogs initially treated only with anti-seizure medications were later given analgesia for HSM.

**Figure 3:**
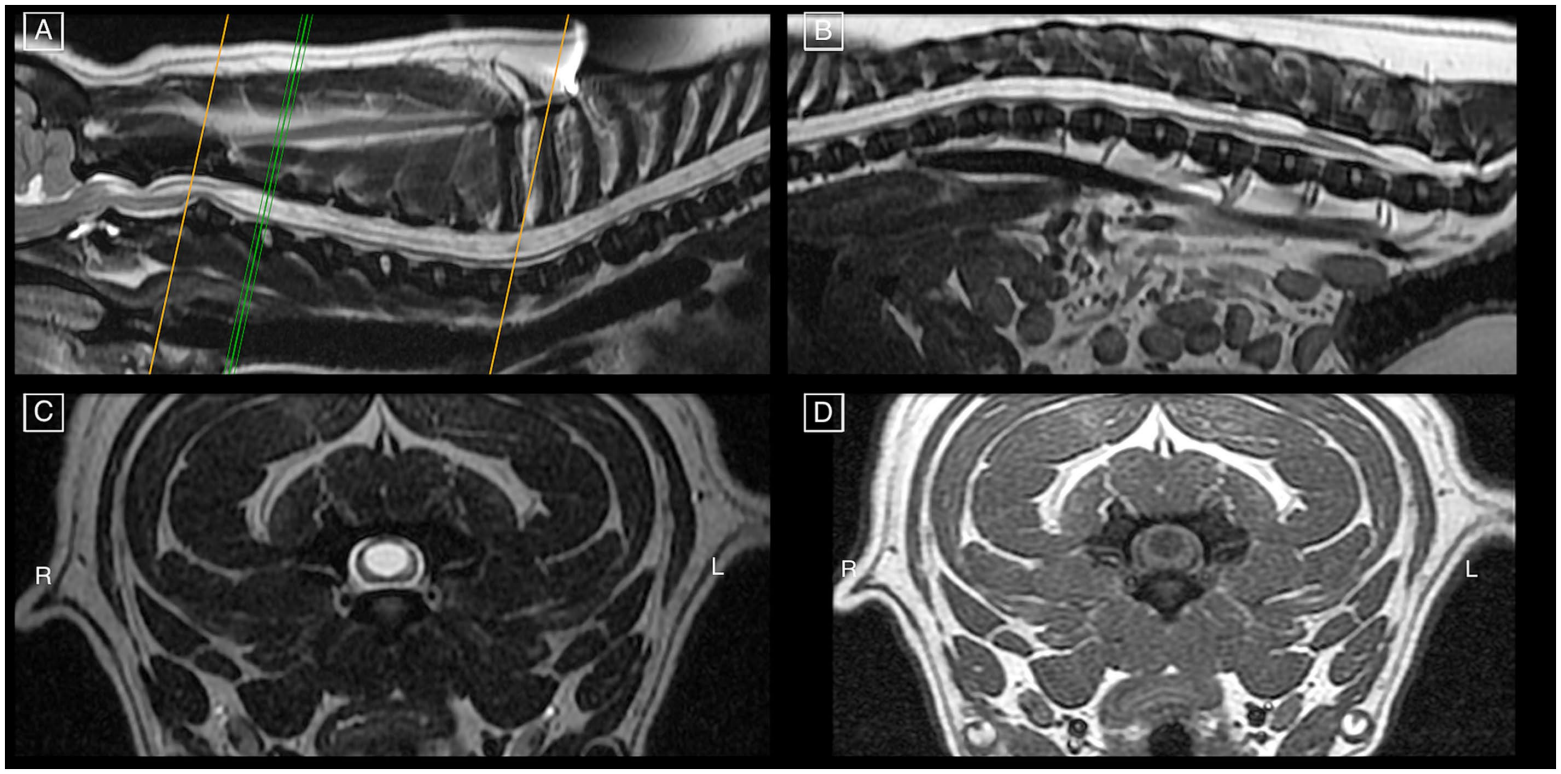
Pre-operative MRI study of a French Bulldog on mid-sagittal T2w images **(A,B)** of all spinal cord segments and cranio-cervical junction, demonstrating features of HSM occupying more than 75% of the spinal cord diameter as seen on transverse T2w **(C)** and T1w **(D)** images at the level of C3-C4. There are signs of CM, mildly increased odontoid angulation, mildly compressive dorsal atlanto-axial band, and intervertebral disk protrusion at C2-C3 with minimal spinal cord compression.

**Figure 4:**
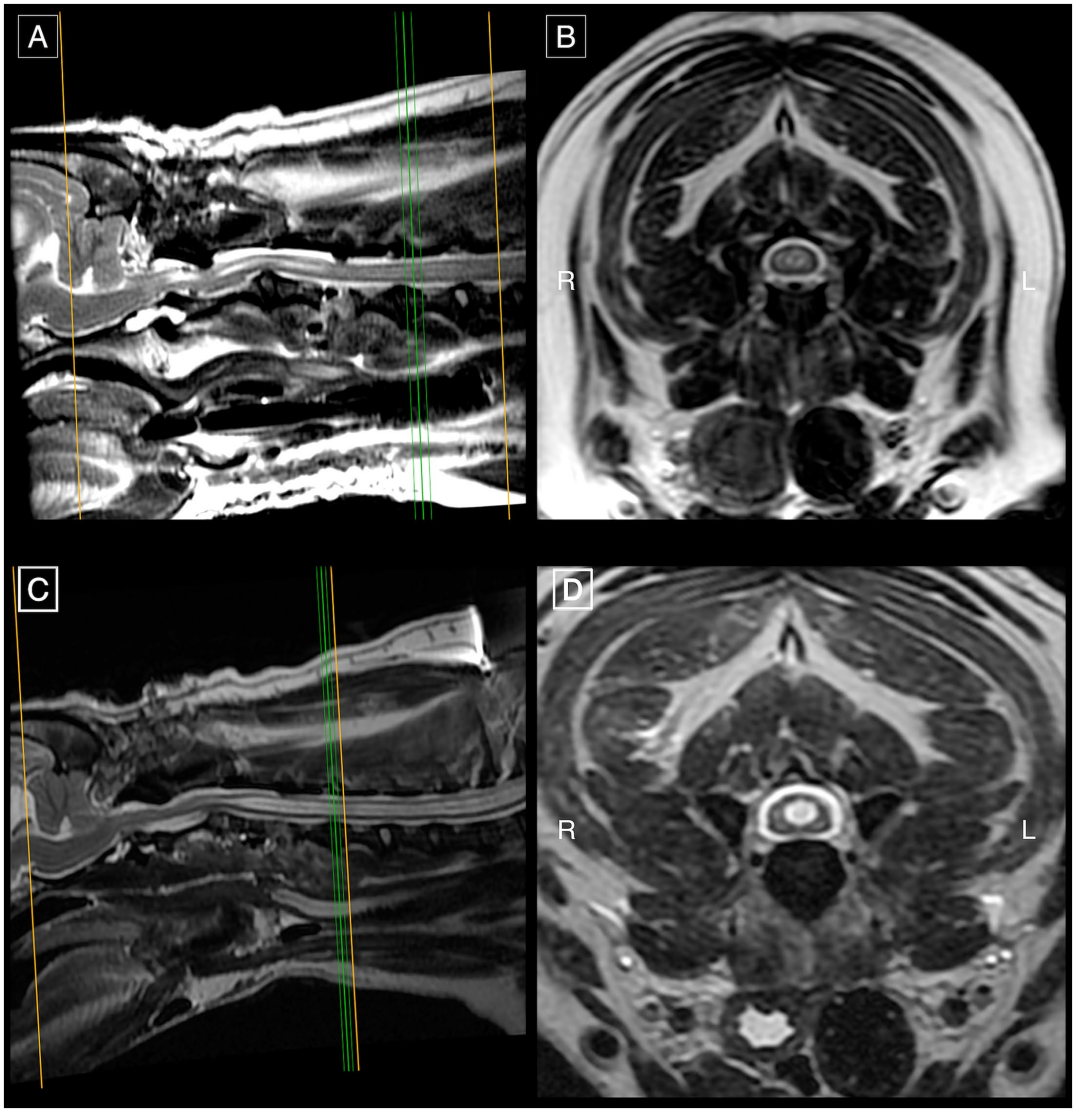
First post-operative MRI study of the same French Bulldog of Figure 3 on mid-sagittal **(A)** and transverse **(B)** T2w images at the level of C4-C5 demonstrating substantially improved SM in the cervical spinal cord segments in comparison to pre-operative SM severity (Figure 3). Second post-operative MRI with mid-sagittal **(C)** and transverse **(D)** T2w images at C4-C5 showing refilled SM in the cervical spinal cord segments with a diameter of similar dimensions to its pre-operative size.

The dog that underwent surgery had a 20-month post-operative follow-up. Owner-reported improvements in myelopathic signs and overall comfort were noted and QoL was 4, though the neurological grade remained unchanged (grade 2). The dog was receiving gabapentin once daily. Immediate post-operative MRI studies were conducted, one after each surgery: the first demonstrated a substantial syrinx reduction, and the second (6 days later) revealed a refilled syrinx with a diameter comparable to its pre-operative size (Figures 3, 4).

Based on the evolution of clinical signs at follow-up in 16 surviving dogs, one dog improved (6%), nine deteriorated (56%), and four remained static (25%). Of the four dogs without clinical signs at presentation, two remained normal, one eventually developed signs attributed to HSM, and one was deceased.

## Discussion

To the authors’ knowledge, this is the first study focusing specifically on the holocord variant of canine SM. We described the clinical and MRI findings of HSM and explored this rare form of an otherwise extensively studied condition. Providing a first description of the clinical course of HSM could assist clinicians in improving their understanding of this uncommon spinal cord disorder. It could also create the basis for further research into this currently poorly understood and severe SM type. In human medicine, HSM also requires further study, with only scarce reports identified in the literature (23–26).

French Bulldogs accounted for an average of 4.8% of the total canine population across the three participating hospitals, yet they comprised half of our cohort. In contrast, CKCS made up 11% of the study population. This disproportionate inclusion of French Bulldogs contrasts with existing literature suggesting that other breeds are more commonly affected by SM (1, 3, 6, 7, 21, 35–37). For instance, the prevalence of clinical SM in CKCS at 6 years of age was estimated at 15% (37), while 24% of Pomeranians and 52% of Griffon Bruxellois dogs have been reported to have SM (3, 7). Conversely, Ricco et al. found that only 8% of FBs requiring brain or cervical spinal cord imaging had SM (6). Similarly, another study documented SM in only 2.7% of FBs with myelopathy (36). However, more recent literature indicates a significantly higher prevalence of SM in FBs (38). The overrepresentation of FBs in our cohort does not imply a high incidence of HSM in the breed but rather points to a potential breed predilection for this type of SM. Future studies should investigate HSM in overrepresented breeds, as this could help uncover potential genetic factors and their developmental mechanisms contributing to this complex pathology.

One explanation for the continuous HSM pattern is the progression and merging of multifocal syringes, as documented in a CKCS diagnosed with SM at 8 months, advancing to HSM by 3 years (11). In our study, HSM was diagnosed at first MRI in all cases, with a median delay of 7 months from clinical onset, potentially hindering early detection of pre-HSM stages. In humans, congenital idiopathic central canal dilatation is reported without clinical signs and with a static radiological appearance over time (39, 40), a finding that contrasts with HSM in both clinical relevance and MRI features. However, it remains unclear whether subclinical dogs with incipient SM may initially resemble this form long before progressing to HSM. Longitudinal studies with sequential MRIs over a median interval of 6 years did not show progression from SM to its holocord variant (22), suggesting distinct mechanisms of development. The recognition of emerging SM stages before holocord severity is further complicated by its occurrence without associated clinical signs (35), indicating that some cases in our cohort may have had subclinical SM for years. The median age of clinical onset for SM in CKCS is 5 years (41), with signs rarely developing after the age of six (37). The median age at presentation in our cohort was nearly 7 years, which presumably allows sufficient time for SM to manifest clinically before advancing to HSM. Yet, this difference in age at clinical onset may reflect the underrepresentation of CKCS in our population. Ultimately, our study was not designed to evaluate the longitudinal progression of HSM, and definitive conclusions about its etiopathogenic distinction from CM-SM or potential parallels with human variants of SM cannot be drawn. Future research should explore disparities between HSM and CM-SM, including clinical and imaging characteristics identified in our study, as well as the role of congenital malformations, particularly in light of recent reports of SM occurring independently of CM or brachycephaly (38).

In our study, more dogs exhibited signs of myelopathy than spinal pain/paresthesia/allodynia. In CKCS with CM/SM, pain is more prevalent than myelopathy (10, 20), with the latter linked to a syrinx width of 4 mm or more (20, 34). Nalborczyk et al. reported an association between a large syrinx in the dorsal horn of the mid-cervical spinal cord and phantom scratching (42), though this was not reproducible in another study (20). In FBs with cervical SM, proprioceptive deficits (83%), ataxia (67%), and neck pain (42%) are most frequent (6). Similarly, in our FB cohort, 78% presented signs of myelopathy attributed to HSM and 33% signs of spinal pain/paresthesia/allodynia. In contrast, Rusbridge and colleagues found neurological abnormalities of myelopathy in only 35% of CKCS with CM and SM (20). The presence of myelopathic signs may correlate with SM severity, particularly syrinx width, though this parameter was not assessed in our cases. Notably, myelopathic signs were less common (50%) in non-FB dogs despite the severely extensive HSM. It is important to recognize that French Bulldogs are predisposed to other spinal conditions, including IVDE, spinal arachnoid diverticulum, incidental vertebral malformations, as well as inflammatory and neoplastic disorders (36). Concurrent spinal conditions were documented and the prevalence of myelopathic or pain-related signs attributed to HSM was calculated after exclusion of other possible causes.

Nine dogs were referred with clinical complaints unrelated to HSM, though five of them had associated neurological signs detected upon examination. This may be due to factors such as pet owners dismissing or normalizing certain neurological signs or not seeking specialist advice for mild manifestations. Additionally, HSM might present in a more benign form in some cases, lacking debilitating or acute neurological complications, and thus not requiring urgent specialist involvement. In contrast to our findings, SM is frequently observed without associated clinical signs, with the proportion of clinically affected dogs increasing with age (35, 41, 43), as noted in one of four initially normal dogs in our cohort. Our methodology was not intended to assess the prevalence of HSM in a broader population of clinically normal dogs; thus, the incidence of non-clinical HSM may be higher than currently recognized, notwithstanding its apparent severity on MRI.

Holocord SM is thought to extend from C2 to L4 vertebrae (21), though this assertion lacks supporting published veterinary data. Our findings confirm that the cranial limit of HSM is more commonly at C2, while the caudal limit varied from L3 to L6. Another parameter of increasing scientific interest in veterinary literature is the termination level of the conus medullaris. In our cohort, the conus medullaris predominantly terminated at L6. A recent study found that a caudal termination level of the conus medullaris, but not of the dural sac, was associated with lumbar SM in CKCS (44). Our findings contradict this observation, as HSM was present despite a more cranial conus medullaris termination in some dogs. This discrepancy suggests that a different mechanism might contribute to HSM development. Additionally, CKCS are known to have a more caudal spinal cord and dural sac termination (45), though the significance of this remains unclear. Generally, the level of termination of the conus medullaris is influenced by body weight (46), but this correlation was not assessed in our study population.

In our cohort, MRI revealed CM and/or other intracranial or craniocervical malformations in all but one dog. Knowler et al. identified a reduced occipital crest and cervical flexure as craniometric traits linked to SM in certain breeds (47), though other studies failed to predict SM progression based on craniocervical junction morphology (48). Odontoid angulation with craniocervical junction elevation (medullary kinking) was detected in some of our cases and contributed to cervical flexure development, a change in angulation compromising neural parenchyma compliance and CSF circulation dynamics (47). Although canine CM is often reported in association with SM, its severity was mild in most of our cases, and some dogs lacked CM entirely, suggesting that it is not a prerequisite for HSM development. Hoholm et al. found SM without CM in 17 of 163 dogs, supporting alternative pathogenic mechanisms (38). Another abnormality observed in our cohort was the presence of atlanto-axial bands, although its prevalence was not as high as in middle-aged to older CKCS, where increased compression correlates with more advanced SM (49). The severity of atlanto-axial bands on MRI should be interpreted cautiously, as spinal cord compression can worsen with neck extension (49) or an endotracheal tube tie over that region (50). A more frequent MRI finding was CSF flow artifact, potentially indicating turbulent or pulsatile CSF flow, which may contribute to syrinx expansion (11). Finally, nearly all cases exhibited ventriculomegaly, though its relationship with HSM remains unclear, as no association between ventriculomegaly and SM clinical severity has been established (51).

The pathogenesis of SM has been extensively studied; however, its association with specific developmental anomalies remains uncertain (12). Earlier research largely focused on caudal cranial fossa overcrowding as the primary etiological factor. More recently, attention has shifted to the concept of brachycephalic obstructive CSF channel syndrome (BOCCS), which entails more complex etiopathogenic hypotheses. Suggested mechanisms involve osseous and neural parenchymal reduction and displacement, leading to disruptions in CSF circulation and absorption, ultimately predisposing to SM development (12). BOCCS is associated with notable malformations, including reduced interparietal and supraoccipital bones, increased cranial and occipital lobe height, a diminished skull base with spheno-occipital synchondrosis angulation and sphenoid flexure, reduced caudal fossa volume with cerebellar vermis impaction/herniation into the foramen magnum, rostral displacement of the atlas and axis with odontoid elevation contributing to cervical flexure, olfactory bulb rotation and reduction, and frontal lobe flattening (11, 12). In our cohort, 16 out of 18 dogs were brachycephalic. Although comprehensive evaluation of these abnormalities was limited due to incomplete brain MRI studies in some patients, many of the aforementioned features were observed in our brachycephalic group. Conversely, two dogs in the current study had no CM and seven had only supraoccipital cerebellar indentation without cerebellar vermis foramen magnum impaction/herniation (CM grade 1). Moreover, HSM was identified in one dolichocephalic and one mesocephalic breed. These findings align with recent literature reporting SM in non-brachycephalic breeds and in the absence of CM (38). As a result, the pathogenesis of SM and its holocord variant warrants further investigation through studies employing more objective morphometric analyses and comparative statistical assessments.

Gabapentin was the most frequently used medication, and polytherapy was common. Corticosteroids were also often prescribed. Gabapentin has been shown to be effective for neuropathic pain secondary to CM-SM when combined with NSAIDs (52). Pregabalin also improves clinical signs caused by SM in CKCS (53, 54) as do anti-inflammatory medications, corticosteroids or NSAIDs (55). In our study, the evolution of neuropathic pain was overall negative despite medical therapy which contradicts the above observations. Further research is needed on the efficacy of various pharmaceutical options, as clinical SM can motivate euthanasia in 15–20% of affected dogs (37, 56). Although none of our cases with follow-up were euthanased because of HSM, our median follow-up duration was relatively short, preventing conclusions about the long-term impact of HSM on survival.

In the current study, one dog underwent surgery using a technique that has shown excellent outcomes in people with CM-I and associated HSM (57). Most dogs undergoing foramen magnum decompression for CM-SM have shown improved clinical outcomes and QoL (58–60), despite an unchanged syrinx size in some cases (61). Post-operative treatment varied across studies, with some dogs continuing medical therapy (60), and others requiring sporadic medication (58, 59). In our case, the first post-operative MRI showed significant syrinx reduction, while the second revealed a refilled syrinx of similar diameter. Despite persistent SM and an unchanged neurological grade, subjective improvements in clinical signs and QoL were reported. However, this lacked objective validation, and the dog remained on medication at follow-up. Further research is needed to clarify long-term outcomes and relapse risks following surgery, as well as to determine whether surgery is superior to medical treatment.

An unchanged neurological grade was maintained in most cases. Although more than 50% exhibited clinical deterioration, including worsening myelopathy in eight dogs, none progressed to a non-ambulatory status. One dog with no myelopathy at presentation later developed such deficits. Spinal pain/paresthesia/allodynia worsened in seven dogs, including four that initially lacked these signs but developed them at follow-up. Plessas and colleagues reported that 75% of CKCS with SM showed progressive signs, with most owners rating their dogs’ QoL as satisfactory (56). Many dogs in our study had a subjectively positive QoL rating 3 or above, although long-term medical therapy was required in most, including the dog treated surgically. The relatively short follow-up period limits our understanding of long-term prognostic implications and treatment requirements. More time may be needed for significant neurological deterioration to occur with HSM.

This study has several limitations. Its multicentric, retrospective design may have influenced data collection and interpretation, potentially affecting the evaluation of clinical signs and their correlation with identified diagnoses. Additionally, the possibility of overlapping signs from concurrent conditions involving the same region cannot be excluded. Moreover, the small sample size limits our ability to draw any robust conclusions about the clinical and imaging features of HSM. Follow-up data were not uniformly supported by re-examinations performed by a neurologist; only six cases received objective neurological assessments at follow-up. Nonetheless, data from the remaining 10 dogs were supplemented with video recordings. Variability in MRI protocols further constrained the characterization of imaging findings and prevented us from performing detailed morphometric analysis. Non-standardized and non-randomized treatment approaches preclude the evaluation of specific therapeutic efficacy. Finally, the median follow-up duration of 12 months is insufficient to describe the long-term clinical course of the disorder.

## Conclusion

This study provides the first focused characterization of the holocord variant of SM in 18 dogs, describing its clinical and imaging features and discussing gaps in our understanding of its potential pathophysiology. Notably, the study highlights a breed predilection, with French Bulldogs representing half of the cohort. Despite the small sample size and the retrospective nature of this study, our findings suggest that HSM presents with myelopathy in most cases, particularly in French Bulldogs, and often leads to clinical progression. The long-term prognosis remains unclear due to the relatively short follow-up period, and further studies are required to clarify potential disparities with the well-described SM-CM phenotype. Future research should investigate the genetic and developmental factors underlying HSM, refine diagnostic and screening criteria, and evaluate therapeutic interventions to enhance long-term outcomes.

## Data Availability

The original contributions presented in the study are included in the article/Supplementary material, further inquiries can be directed to the corresponding authors.
