# Supplementary material for: Holocord syringomyelia in 18 dogs
Source: Front Vet Sci. 2025 Jan 8;11:1514441. doi: 10.3389/fvets.2024.1514441 (PMC11750772; doi:10.3389/fvets.2024.1514441)
Supplement: Supplementary file 1 [file Data_Sheet_1.pdf]

frontiers

Supplementary Material

Holocord Syringomyelia in dogs: clinical and MRI findings

Follow-up Questionnaire:

**Patient Name**	
**Patient ID**	
**Referral Centre**	
**Date of diagnosis**	
**Date questionnaire completed**	

1. How do you perceive your dog's **overall quality of life** at present (1 being very poor, and 5 being excellent)?

   ☐ 1
   ☐ 2
   ☐ 3
   ☐ 4
   ☐ 5
   ☐ Prefer not to say
   ☐ Difficult to say – I do not know

2. Following discharge from <<INSTITUTION>>, how do you feel your dog's **neurological signs** have changed if at all?

   ☐ Marked improvement - back to normal

   ☐ Some improvement - much better but some disability remaining

   ☐ Little or no improvement

   ☐ Some deterioration – mild

   ☐ Significant deterioration

   ☐ Difficult to say – I do not know

3. Is he/she receiving any **medications** at the moment?

Yes	
No	
I do not know	

If yes, can you provide details about the name of the medication, the dose, how often he/she receives it and for how long?

Name	Dose	Frequency	Duration of treatment	Condition treated with this medication

4. How do you feel your dog's neurological signs have changed, if at all, after introduction of the above **medications**?

☐ Marked improvement - back to normal

☐ Some improvement - much better but some disability remaining

☐ Little or no improvement - static

☐ Some deterioration – mild

☐ Significant deterioration

☐ Difficult to say – I do not know

5. Have you noticed any **changes in the gait** (manner of walking) of your dog in comparison to his/her gait at the time of discharge from <<INSTITUTION>>?

☐ Marked improvement - back to normal

☐ Some improvement - much better but some disability remaining

☐ Little or no improvement - static

☐ Some deterioration – mild

☐ Significant deterioration

☐ Difficult to say – I don't know

☐ No gait abnormalities at discharge or at present

Please describe current gait abnormalities:

6. Have you noticed any **changes in the levels of pain or discomfort** in comparison to the time of discharge from <<INSTITUTION>>?

☐ Marked improvement - back to normal

☐ Some improvement - much better but some disability remaining

☐ Little or no improvement - static

☐ Some deterioration – mild

☐ Significant deterioration

☐ Difficult to say – I do not know

☐ No pain/discomfort at discharge or at present

Please describe current signs of pain/discomfort:

7. Have you observed any of the following **specific signs**?

	**Yes**	**No**	**I don't know**	**Details**	**Was this sign present at discharge? (Yes/No)**	Has this sign **improved**/**deteriorated**/remained **unchanged** since discharge?
Example clinical sign	*X*			*In the right front limb*	*Yes*	*Deteriorated*
Thoracolumbar Kyphosis (arched back)						
Cervical Scoliosis (twisted neck)						
Thoracolumbar Scoliosis (twisted back)						
Thoracolumbar Lordosis (excessive downward curve of the back)						
Head tilt						
Low head/neck carriage						
Knuckling while standing/walking						
Episodes of yelping/crying						
Difficulty moving the neck						
Scratching behaviour						
Head shaking						
Rubbing of the face						
Being withdrawn						
Refusal to exercise						
Excessive licking/chewing of any of the paws						

Falling while walking/running						
Knuckling/scuffing while walking/running						
Weakness while walking/running						
Lameness while walking/running						
Incoordination/wobbliness (crossing, abduction, stumbling, etc.) while walking/running						
Exercise intolerance						
Loss of balance while walking/running						
Other – specify						

8. Can you please provide us with a video recording of your dog walking?

Yes	
No	

Please forward the video recordings to the following email address:
despoina.douralidou@theralph.vet
